# Non-pharmacological interventions for sleep in older adults: an umbrella review and evidence map of randomized controlled trials

**DOI:** 10.3389/fneur.2025.1655192

**Published:** 2025-09-02

**Authors:** Yawei Yu, Huifeng Wang, Wei Li, Hong Guo, Yiping Chen

**Affiliations:** ^1^School of Nursing, Beijing University of Chinese Medicine, Chaoyang, Beijing, China; ^2^Peking Union Medical College Hospital, Dongcheng, Beijing, China

**Keywords:** non-pharmacological, sleep, therapies, treatment, aged, umbrella review

## Abstract

**Background:**

Sleep health is a critical determinant of older adults’ physical, cognitive, and emotional well-being, yet pharmacological treatments for sleep disturbances carry substantial risks.

**Objective:**

This umbrella review aims to synthesize evidence on the effectiveness of non-pharmacological interventions (NPIs) for improving sleep in older adults, to inform clinical decision-making and future guidelines.

**Methods:**

This review adhered to PRIOR and PRISMA guidelines and was registered in PROSPERO (CRD42024565849). Systematic searches were conducted across six databases from inception to July 6, 2024. Eligible studies were systematic reviews with meta-analyses of randomized controlled trials (RCTs) targeting adults aged ≥60 years. Two reviewers independently screened studies, extracted data, and assessed methodological quality using AMSTAR 2. Certainty of evidence was rated with GRADE, and review overlap was quantified using the Corrected Covered Area (CCA). Narrative synthesis was conducted due to high heterogeneity.

**Results:**

Nineteen systematic reviews comprising 160 RCTs were included. Interventions covered six categories: cognitive behavioral therapy (CBT), mindfulness, exercise (e.g., Tai Chi), music, manual therapies (e.g., massage, acupuncture), and joint approaches. CBT significantly improved sleep onset latency (−9.29 min), wake after sleep onset (−22 min), and sleep efficiency (+7.9%). Exercise, particularly Tai Chi, reduced PSQI global scores by −1.05. Music and manual therapies also showed benefits, though with inconsistent effect sizes. Most reviews were of low methodological quality, and the certainty of evidence ranged from low to very low. CCA was 3.68%, indicating slight overlap.

**Conclusion:**

CBT and exercise-based interventions are promising for improving sleep in older adults. However, the certainty of evidence remains limited. Future high-quality RCTs are needed, and the evidence map highlights priority areas for research in geriatric sleep health.

**Systematic review registration:**

https://www.crd.york.ac.uk/PROSPERO/, identifier CRD42024565849.

## Introduction

The prevalence of sleep disorders among the elderly is notably high. A recent systematic review (1) indicated that nearly 50% of individuals aged 60 and above worldwide report varying degrees of sleep disturbances. These issues include poor sleep quality (47.12%), short sleep duration (40.81%), long sleep duration (31.61%), and insomnia (21.15%). Such sleep disorders significantly impact the overall health and quality of life of older adults (2, 3). Promoting sleep health in this population is crucial, as sleep disorders are associated with various adverse health outcomes, including cognitive decline, mood disorders, depression, cardiovascular diseases, diabetes, and increased mortality risk (4–7). Addressing sleep problems in the elderly can enhance their physical and mental health, improve daily functioning, and elevate their quality of life.

Several studies have investigated the effects of non-pharmacological interventions on sleep in older adults. For instance, a systematic review and meta-analysis explored the impact of different meditation practices on the sleep quality of the elderly (8). Social activity intervention (9) have also been examined for their potential to enhance sleep quality among older adults. Additionally, research on the effects of mindfulness-based cognitive therapy (10) on sleep disorders in the elderly has shown positive outcomes in improving sleep quality. However, to date, most systematic reviews and meta-analyses (10–13) on sleep health interventions for the elderly have focused on single treatment modalities. Although network meta-analyze (14) has compared various non-pharmacological sleep interventions, the results are limited in clinical application due to the heterogeneity of the included evidence, indirectness of the analyses, and issues of heterogeneity and transferability in specific clinical settings.

An umbrella review can comprehensively integrate evidence from systematic reviews. Currently, there is no comprehensive review and analysis of meta-analyses addressing the effects of all non-pharmacological interventions on sleep in the elderly. Conclusive evidence on which non-pharmacological intervention is most effective in promoting sleep health in older adults remains lacking. This review aims to thoroughly examine existing evidence, evaluate non-pharmacological interventions that promote sleep health in the elderly, and consider the effect size, quality, and certainty of the evidence to provide a basis for future guidelines. Additionally, we will generate an evidence map on the effectiveness of various non-pharmacological interventions in improving sleep among older adults.

## Methods

### Reporting guidelines

This umbrella review complied with the Preferred Reporting Items for Systematic Reviews and Meta-Analyses (PRISMA) checklist (15) and was registered in the International Prospective Register of Systematic Reviews (PROSPERO; registration number: CRD42024565849).

### Search strategy and study selection

PubMed, Embase, the Cochrane Database of Systematic Reviews (Ovid CDSR), PsycINFO, CINAHL, and Scopus were systematically searched from inception to July 6, 2024. Manual searches of the reference lists of all eligible systematic reviews were also conducted. The complete search strategy is provided in the Supplementary materials. The search terms were selected based on the target intervention (i.e., NPIs), population (i.e., older adults), and outcomes (i.e., sleep), using database-specific indexing syntax. Consensus on the final keywords was reached by all authors. Title/abstract and full-text screenings were performed independently by multiple reviewers, with any discrepancies resolved by a third reviewer.

### Eligibility criteria

The Inclusion criteria follow the PICOs framework of patients (P), interventions (I), outcomes (O), and study design (S):

1. Population: Older adults aged ≥60 years, with or without sleep disorders;
2. Intervention: Systematic reviews with meta-analyses of RCTs involving at least one non-pharmacological intervention (e.g., exercise therapy, cognitive behavioral therapy, mindfulness, music therapy, acupuncture, light therapy, aromatherapy, meditation, or mind–body therapy);
3. Study design: Systematic reviews including RCTs;
4. Comparison: Control group received a waiting list, usual care(e.g., health education, sleep hygiene advice), placebo, or another non-pharmacological intervention(e.g., sleep duration, sleep quality);
5. Outcomes: Sleep-related outcomes reported as primary or secondary endpoints.

Exclusion criteria included case studies, narrative reviews, systematic reviews without meta-analyses, and reviews using pharmacological comparators. Reviews published in languages other than English were also excluded due to limitations in database access. No restrictions were placed on publication year.

### Data extraction

Two reviewers (YP and LW) independently extracted data on: first author, year of publication, country, mean age, gender, intervention type, outcomes, number of RCTs, total participants, between-study heterogeneity, and summary effect estimates. Disagreements were resolved through discussion or consultation with a third reviewer (HF).

### Quality assessment of included reviews

The methodological quality of the included reviews was assessed using AMSTAR 2, a validated tool for evaluating systematic reviews of healthcare interventions. The risk of bias (RoB) for individual RCTs was extracted from the most recent and highest-quality review available, based on standard domains such as random sequence generation, allocation concealment, blinding, incomplete outcome data, selective reporting, and other potential biases. If no review rated as moderate or above was available, the RoB was independently evaluated by two reviewers.

### Data synthesis and certainty of evidence

The degree of overlap in RCTs across included reviews was assessed using the Corrected Covered Area (CCA) method (14). A CCA of 0% indicates no overlap; values were interpreted as follows: 0–5% (slight), 6–10% (moderate), 11–15% (high), and >15% (very high overlap).

Included reviews were narratively synthesized based on intervention type, key findings, and methodological quality. We did not reanalyze original RCTs; instead, we extracted effect sizes and 95% confidence intervals from the meta-analyses (16). When both fixed-effect and random-effects models were reported, the random-effects results were prioritized. I^2^ and *p*-values from Egger’s or Begg’s tests were used to assess heterogeneity and publication bias. Due to the high I^2^ (>50%) in most included meta-analyses, pooled analyses were not conducted, and findings were reported narratively.

An evidence map was created to visually present the benefits or harms, evidence certainty, and research gaps for each intervention. Certainty of evidence was assessed using the GRADE (Grading of Recommendations Assessment, Development, and Evaluation) framework and categorized as high, moderate, low, or very low (17). All evaluations were conducted independently by two reviewers (YP and LW), with oversight from a third reviewer (HF).

## Results

### Search results and characteristics of included studies

**Figure 1:**
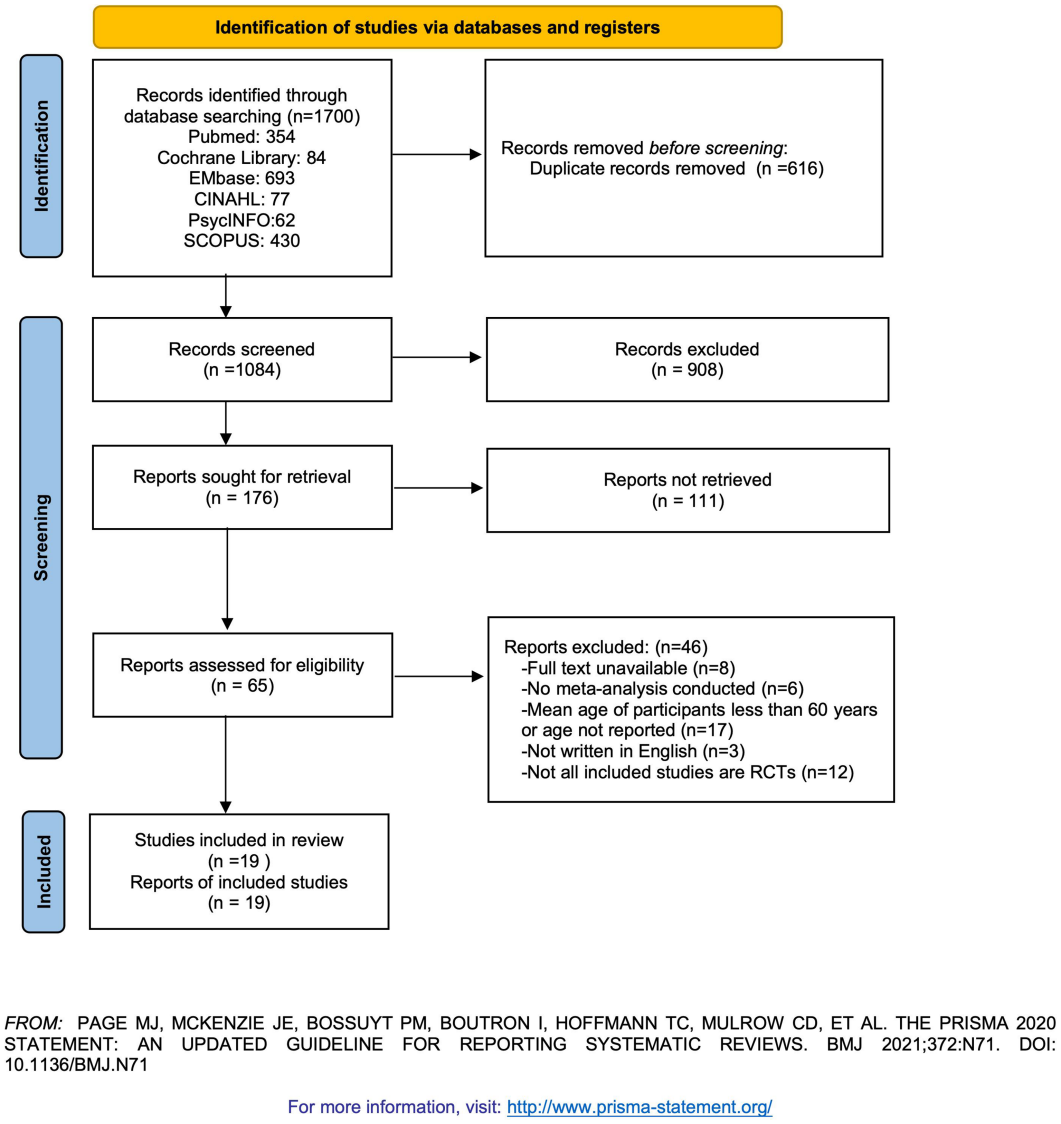
PRISMA diagram of the literature search.

A total of 1,700 records were identified through database searches (PubMed: 354; Cochrane Library: 84; Embase: 693; CINAHL: 77; PsycINFO: 62; SCOPUS: 430). After duplicates were removed, 1,084 unique records remained. Following title and abstract screening, 65 records were retained for full-text review (Figure 1). During the full-text screening, 46 records were excluded. References for the excluded studies are provided in Supplementary Table S2. Ultimately, 19 eligible meta-analyses (references in the Supplementary material) were included, encompassing 160 RCTs. Six types of non-pharmacological interventions were reported, including Cognitive Behavior Therapy, Mindfulness Therapy, Exercise Therapy, Music Therapy, Manual Therapies (Acupressure, Acupuncture, Massage Therapy, or Aromatherapy), and Joint Interventions (definitions for each intervention are provided in Supplementary Table S3). The basic characteristics and intervention outcomes of the included studies are presented in Tables 1, 2. The CCA of the included studies was 3.68%, which is less than 5%, indicating slight overlap.

**Table 1:** Characteristics of included reviews.

Authors, year	Type of review	Objective	No. databases	Included RCT studies	Type of intervention	Countries
Montgomery et al. (18)	SR and MA	To assess the efficacy of cognitive-behavioral interventions in improving sleep quality, duration and efficiency among older adults (aged 60 and above)	5	6 (5 included in meta-analyses)	Cognitive-behavioral interventions	5 USA 1 Canada
Yang et al. (26)	SR and MA	To identify the efficacy of an aerobic or resistance exercise training program in improving sleep quality in middle-aged and older adults with sleep problems	6	6 (5 included in meta-analyses)	Exercise therapy	6 USA
Du et al. (27)	SR and MA	To examine the efficacy of Taichi exercise in promoting self reported sleep quality in older adults	8	5	Taichi exercise	2 USA 1 Germany 1 Iran 1 China
Wu et al. (28)	SR and MA	To identify the effect of meditative movement interventions on older people’s quality of sleep.	8	14 (12 included in meta-analyses)	Meditative movement interventions	5 USA 4 China 2 India 1 Japan 1 Iran 1 Germany
He et al. (8)	NMA	To estimate the efects of diferent meditation exercises on the improvement of sleep disorders in older people	2	10	Meditation exercises	5 USA 4 China 1 India
Chen et al. (33)	SR and MA	To examine the effects of acupressure on the health promotion in older adults	11	18	Acupressure	1 USA 16 China 1 Spain
Chen et al. (11)	SR and MA	To identify the effect of listening to music on sleep quality in older adults	5	5	Listening to music	3 China 2 Singapore
He et al. (44)	SR and MA	To assess the effects of traditional Chinese exercises and general aerobic exercises on the sleep quality of older adults	8	22	Traditional Chinese exercises and general aerobic exercises	6 USA 11 China 1 Japan 1 Iran 1 Germany 1 Turkey 1 Brazil
Dincer et al. (13)	SR and MA	To determine the effect of acupressure on sleep quality in elderly people.	6	11 (10 included in meta-analyses)	Acupressure	10 China 1 Iran
Hasan et al. (30)	SR and NMA	To identify the efficacies of various exercise regimens in improving sleep quality in older adults	6	35	Exercise regimens	12 USA 9 China 2 Spain 2 Turkey 2 Brazil 2 Korea 1 Germany 2 India 1 Iran 1 Vietnam 1 Japan
Huang et al. (19)	SR and MA	To evaluate the efficacy of cognitive behavioral therapy for insomnia (CBT-I) in older adults	6	13	Cognitive behavioral therapy	10 USA 1 Australia 2 Canada
Chen et al. (20)	SR and MA	To evaluate the efficacy of a brief 4-week behavioral therapy for insomnia (BBTi) on insomnia remission in older adults with chronic insomnia	8	4 (3 included in meta-analyses)	A brief 4-week behavioral therapy	4 USA
González-Martín et al. (24)	SR and MA	To analyze the effectiveness of a mindfulness based program on sleep quality in healthy non-institutionalized older people	4	10	A mindfulness based program	3 USA 4 China 1 Spain 2 Singapore
Gu and Lee (21)	SR and MA	To assess the effects of non-pharmacological interventions on sleep in older people	8	15 (10 included in meta-analyses)	Non-pharmacological interventions	1 USA 3 China 3 Turkey 4 Korea 2 Japan 1 Spain 1 Australia
Solis-Navarro et al. (22)	SR and MA	To determine if physical exercise delivered through a structured program improves sleep quality in older adults	4	13 (9 included in meta-analyses)	Structured physical exercise program	2 USA 3 China 2 Turkey 1 Korea 1 Spain 1 UK 1 Tunisia 1 Canada 1 Japan
Chang et al. (23)	SR and NMA	To compare the effectiveness of non-pharmacological interventions in enhancing sleep quality in older people	7	71	Non-pharmacological interventions	12 USA 32 China 3 Korea 1 Tunisia 1 Egypt 2 Turkey 1 Turkiye 3 Australia 3 Brazil 6 Iran 1 Spain 1 Thailand 1 Japan 2 India 1 Germany
Lannon-Boran et al. (25)	SR and MA	To investigate the effect of mindfulness-based intervention (MBI) on cognitively unimpaired older adults’ cognitive function and sleep quality	4	7 (3 included in meta-analyses)	Mindfulness-based intervention	4 USA 3 Canada
Li et al. (31)	SR and MA	To investigate the effects of tai chi on moderate-to-severe elderly patients with sleep disorders	9	7	Tai Chi exercise	2 USA 4 China 1 Iran
Lyu et al. (32)	SR and MA	To investigate the effects of Tai Chi exercise on the sleep quality of older adults living in the community	8	12	Tai Chi exercise	5 USA 6 China 1 Vietnam

**Table 2:** Extracted data from reviews studying results of interventions.

Authors, year	Outcome measure	No. participants included in the meta-analysis	No. intervention group	No. control group	Variation between studies (I^2^)	Review pooled results (95% confidence intervals)
Montgomery et al. (18)	Sleep onset latency as reported in participants’ diaries	135	86	49	0	−3 (−8.92,2.92)
	Wake after sleep onset (WASO) as reported in participants’ diaries	159	95	64	54.94%	−21.84 (−37.3,-6.38)
	Wake after sleep onset (WASO) as measured by polysomnography	59	30	29	0	−24.36 (−41.14,-7.57)
	Sleep duration (total, in minutes) as reported in participants’ diaries	143	76	67	0	−14.56 (−36.13,7.01)
	Sleep duration (total, in minutes) as measured by polysomnography	59	30	29	0	18.93 (−2.74,40.6)
	Sleep efficiency (ratio of time asleep/time in bed) as reported in participants’ diaries	133	86	57	76.96%	−7.49 (−15.45,0.47)
	Sleep efficiency (ratio of time asleep/time in bed) as measured by polysomnography	59	30	29	0	−6.25 (−10.18,-2.31)
Yang et al. (26)	PSQI global score	288			NA	0.47 (0.08, 0.86)
	Subjective sleep quality	239			NA	0.47 (0.20, 0.73)
	Sleep latency	239			NA	0.58 (0.08, 1.08)
	Use of sleep medication	196			NA	0.44 (0.14, 0.74)
Du et al. (27)	PSQI global score	381			68%	−0.87 (−1.25, −0.49)
	Subjective sleep quality	252	135	117	24%	−0.83 (−1.08, −0.57)
	Sleep latency	252	135	117	84%	−0.75 (−1.42, −0.07)
	Sleep duration	134	73	61	0%	−0.55 (−0.90, −0.21)
	Habitual sleep efficiency	252	135	117	28%	−0.49 (−0.74, −0.23)
	Sleep disturbance	252	135	117	38%	−0.44 (−0.69, −0.19)
	Sleep medication	252	135	117	87%	−0.51 (−1.25, 0.23)
	Daytime dysfunction	252	135	117	0%	−0.34 (−0.59, −0.09)
Wu et al. (28)	PSQI global score	856	439	417	70%	−0.70 (−0.96, −0.43)
He et al. (8)	PSQI global score	1,304	665	639	22%	−0.63 (−0.79, −0.47)
Chen et al. (33)	PSQI global score	0	NA	NA	68.73%	0.85 (0.49, 1.22)
Chen et al. (11)	PSQI global score	288	142	146	67%	−1.96 (−3.23, −0.69)
He et al. (44)	PSQI global score	1,563	797	766	93%	−2.34 (−3.14, −1.54)
Dincer et al. (13)	PSQI global score	660	332	328	91%	−1.71 (−2.31, −1.11)
Hasan et al. (30)	PSQI global score	3,519	NA	NA	NA	NA
Huang et al. (19)	Sleep efficiency	778	446	332	77%	8.36 (5.96, 10.76)
	Sleep onset latency	712	410	302	64%	−9.29 (−13.62, −4.96)
	Wake after sleep onset	748	428	320	85%	−23.44 (−32.41, −14.47)
	Total sleep time	619	340	279	63%	−12.35 (−21.27, −3.42)
Chen et al. (20)	PSQI global score	136	67	69	0	−1.07 (−1.43, −0.71)
	Total sleep time measured by actigraphy	141	71	70	0	−25.71 (−40.66, −10.76)
	Wake after sleep onset measured by actigraphy	141	71	70	0	−9.49 (−16.02, −2.96)
	Sleep efficacy measured by actigraphy	141	71	70	0	3.48 (1.49, 5.47)
	Sleep onset latency measured by actigraphy	141	71	70	0	−9.24 (−14.00, −4.47)
	Total sleep time measured by sleep diary	176	88	88	0	−9.65 (−31.81, 12.51)
	Wake after sleep onset measured by sleep diary	176	88	88	0	−20.67 (−30.42, −10.92)
	Sleep efficacy measured by sleep diary	176	88	88	0	7.40 (4.82, 9.98)
	Sleep onset latency measured by sleep diary	176	88	88	0	−20.07 (−27.30, −12.84)
González-Martín et al. (24)	Insomnia Severity Index	370	189	181	5%	−0.326 (−0.471, −0.181)
	PSQI global score	616	310	306	3%	−0.343 (−0.456, −0.229)
	Sleep Onset Latency evaluated by polysomnography	207	103	104	2%	−0.344 (−0.425, −0.263)
Gu and Lee (21)	Pittsburgh Sleep Quality Index and Insomnia Severity Index	712	362	350	92%	1.00 (0.16, 1.85)
Solis-Navarro et al. (22)	PSQI global score	1,624	802	822	97%	−2.49 (−3.84, −1.14)
	Sleep quality by duration time	1,662	822	840	95%	−1.17 (−1.79, −0.54)
Chang et al. (23)	PSQI global score; Insomnia Severity Index (ISI) and Epworth sleepi ness scale (ESS)	7,829	NA	NA	NA	
Lannon-Boran et al. (25)	PSQI global score	394	194	200	54%	−0.92 (−1.77, −0.07)
Li et al. (31)	PSQI global score	589	292	297	48%	−0.6 (−0.77, 0.44)
	Subjective sleep quality	233	121	112	39%	−0.79 (−1.06, −0.52)
	Sleep latency	448	226	222	73%	−0.80 (−1.21, −0.40)
	Sleep duration	135	69	66	8%	−0.38 (−0.72, −0.40)
	Habitual sleep efficiency	233	121	112	0	−0.58 (−0.84, −0.31)
	Sleep disturbances	233	121	112	36%	−0.51 (−0.78, −0.25)
	Sleep medication	233	121	112	0	−0.25 (−0.51, −0.01)
	Duration of dysfunction	233	121	112	0	−0.33 (−0.59, −0.07)
Lyu et al. (32)	PSQI global score	1,170	612	558	91.60%	−1.96 (−3.02, −0.90)

### Quality assessment

Among the 19 included meta-analyses, 94.7% of the literature search strategies were found to include all four elements of PICO, but only 52.6% had registered a review protocol in advance. It was noted that 89.5% of the studies did not explain the selection criteria for the study designs included in the review, and none reported the sources of funding for the studies included. Only one study failed to report the specific reasons for study exclusion, and all studies employed appropriate methods for the statistical combination of results. Details of other quality assessment items can be found in Supplementary Table S4.

### Results of each intervention

#### Cognitive behavior therapy

A total of five meta-analyses were conducted to study the effects of cognitive behavior therapy (CBT) on sleep in older adults (18–22). However, the sleep indicators used in these meta-analyses varied (sleep onset latency, wake after sleep onset, sleep efficiency, total sleep time, PSQI global score). Three meta-analyses summarized the effect of CBT on sleep onset latency in older adults (18–20). Two of these meta-analyses reported positive results, indicating that CBT improved sleep onset latency by 9.29 and 20.07 min, respectively, with significant differences (19, 20). Similarly, these three meta-analyses also examined the effects of CBT on wake after sleep onset in older adults, finding that CBT interventions reduced wake after sleep onset by about 22 min on average. The effects of CBT on sleep efficiency were discussed in all three meta-analyses, with two showing an average increase in sleep efficiency of about 7.9% (19, 20). Two meta-analyses explored the impact of CBT on total sleep time, finding an average reduction of about 11 min (19, 20). Finally, two meta-analyses summarized the effect of CBT on overall sleep quality (PSQI global score), indicating that CBT improved overall sleep quality with an average effect size of approximately 1.2 (21, 23).

#### Mindfulness therapy

A total of three meta-analyses studied the effects of mindfulness therapy on sleep in older adults (8, 24, 25). The three meta-analyses used a common outcome indicator—overall sleep quality (PSQI global score). Among them, only two meta-analyses showed that mindfulness therapy could improve overall sleep quality, with effect sizes of 0.343 and 0.92, respectively, showing considerable differences (24, 25). Another meta-analysis showed that mindfulness therapy did not improve overall sleep quality (−0.78, 95% CI: −1.60, 0.05) (8).

#### Exercise therapy

A total of 10 meta-analyses studied the effects of exercise therapy on sleep in older adults (21–23, 26–32). All 10 studies conducted meta-analyses of the impact of exercise therapy on overall sleep quality (PSQI global score) in older adults. Exercise therapy improved overall sleep quality, with an average effect size of approximately 1.05. Among them, three meta-analyses explored the effect of Tai Chi on overall sleep quality (PSQI global score) in older adults, with an average effect size of about 1.14 (27, 31, 32).

#### Music therapy

A total of two meta-analyses studied the effects of music therapy on sleep in older adults (11, 21). Both meta-analyses examined the effect of music therapy on overall sleep quality (PSQI global score). Although both showed positive effects, the results varied greatly. One study showed an improvement effect of −1.96 (−3.23, −0.69) (11), while the other showed a result of −0.51 (−0.84, −0.18) (21).

#### Manual therapy

A total of four meta-analyses studied the effects of manual therapy on sleep in older adults (13, 21, 23, 33). All four meta-analyses examined the effect of manual therapy on overall sleep quality (PSQI global score), with an average effect size of approximately 1.00.

#### Joint therapy

Only one meta-analysis studied the effect of joint therapy on sleep in older adults. Joint therapy improved overall sleep quality, with an effect size of 1.18 (23).

#### Evidence map

**Figure 2:**
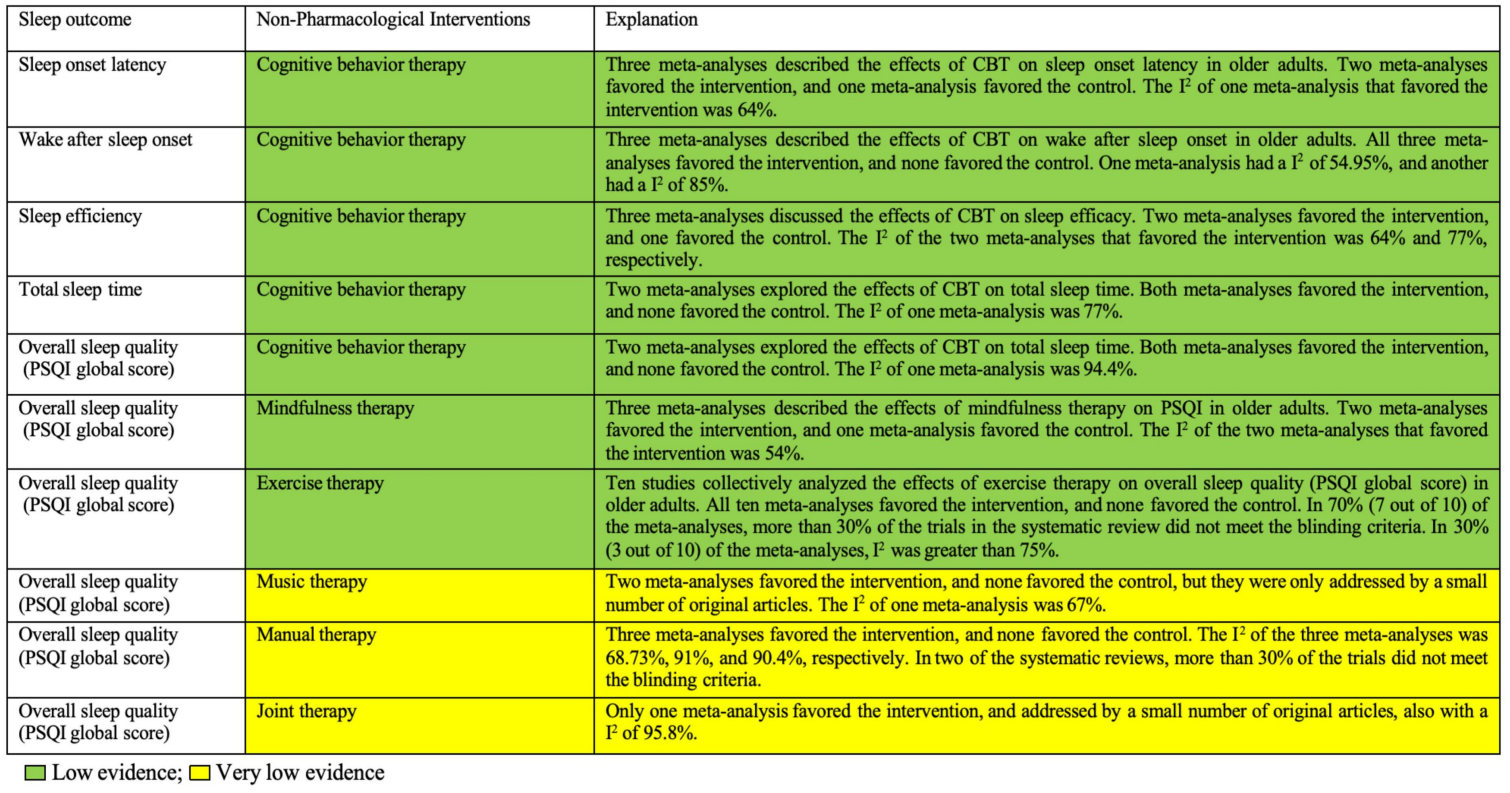
Summary of evidence for the effectiveness of non-pharmacological interventions on sleep outcomes in older adults. CBT, cognitive behavior therapy; PSQI, Pittsburgh Sleep Quality Index.

Figure 2 presents the evidence map summarizing the results of the included interventions. The certainty (or confidence) of the body of evidence on the effectiveness of non-pharmacological interventions for sleep outcomes in older adults is summarized in this paper. The evidence map shows that all non-pharmacological interventions have positive effects on sleep in older adults, but the quality of evidence is classified as low or very low. Specifically, MBT and exercise therapy are graded as low, while the others are classified as very low.

## Discussion

To our knowledge, this is the first umbrella review to comprehensively evaluate the effects of various non-pharmacological interventions on sleep outcomes in older adults. This systematic review analyzed 19 meta-analyses, including a total of 160 RCTs, involving six types of non-pharmacological interventions: CBT, mindfulness therapy, exercise therapy, music therapy, manual therapies, and joint interventions. Manual therapy in this study specifically refers to acupressure, acupuncture, massage therapy and aromatherapy. These findings provide substantial evidence supporting the positive effects of these interventions on various sleep parameters.

Overall, the results of this review indicate consistent evidence that non-pharmacological interventions affect multiple sleep indicators in older adults. Our results show that CBT can significantly reduce sleep onset latency in older adults, by at least 9.29 min; previous study has found (34) that sleep onset latency (>30 min: HR = 1.45, 95% CI: 1.03–2.03) is associated with an increased incidence of dementia in adjusted Cox models. Therefore, the reduction in sleep onset latency by CBT has clinical significance. Similarly, CBT intervention reduced wake after sleep onset by an average of 22 min. Previous research has indicated (35) that increased nighttime awakenings are independently associated with higher mortality risk in older adults. Although our study did not involve the number of awakenings, it nonetheless highlights the importance of wake after sleep onset. In addition, based on our pooled results, CBT increased sleep efficiency by an average of about 7.9%. A prospective cohort study (35) pointed out that low sleep efficiency was nearly significantly associated with mortality in older men, suggesting the importance of sleep efficiency in older adults. Similarly, CBT improved overall sleep quality, with an average effect size of 1.2. A Chinese cohort study (36) also indicated that good/average sleep quality was associated with a 36% lower risk of all-cause mortality in middle-aged and older adults compared to those with a score of 0. From the perspective of improvement in these sleep indicators, CBT carries public health significance.

Among all non-pharmacological interventions, exercise therapy was mentioned most frequently. In this study, all meta-analyses on exercise therapy showed positive effects on sleep improvement, especially Tai Chi, which appeared to be the most effective intervention. Unfortunately, due to many of the included RCTs failing to meet blinding standards, the evidence level was only rated as low. Nevertheless, exercise interventions, as a cost-effective intervention, have significant clinical implications. Exercise therapy not only helps improve sleep quality, but also promotes mental health and reduces the risk of chronic diseases (37, 38). Especially in older populations, exercise therapy can enhance quality of life and independence by increasing physical activity (39). Therefore, future high-quality studies should focus on rigorous trial designs and the application of blinding to improve the reliability and persuasiveness of the evidence. In addition, studies should further explore different types of exercise interventions and their optimal combinations to identify the most suitable strategies for improving sleep in older adults.

Two studies showed that mindfulness therapy significantly improved sleep quality, with effect sizes of 0.343 and 0.92, respectively. However, these effect sizes differ greatly. Another meta-analysis showed that mindfulness therapy did not significantly improve overall sleep quality. This difference in results may stem from various factors. First, the included study samples and quality in different meta-analyses may vary. For example, the study with an effect size of 0.343 included more high-quality RCTs, whereas the study with an effect size of 0.92 included more small-sample studies (three original studies). In addition, the definition and implementation of mindfulness therapy may vary across meta-analyses. Specific intervention methods, duration of therapy, and participant adherence to mindfulness therapy may all affect its effects (40). Furthermore, participant characteristics may also be an important factor. Similar inconsistencies have been found in other studies. Some studies have found (41) that mindfulness therapy significantly improves sleep quality, while others have not reached the same conclusion. To better understand these differences, future research needs to be more systematically and rigorously designed and implemented. At the same time, greater efforts should be made to standardize and refine the specific implementation of mindfulness therapy to ensure the comparability of results. Moreover, researchers should pay more attention to differential responses to mindfulness therapy across populations and explore potential mechanisms and mediating variables.

Music therapy was studied in three meta-analyses, two of which showed positive effects on overall sleep quality. However, the effect sizes differed significantly—one study showed a large improvement (effect size −1.96; 95% CI: −3.23, −0.69), and the other showed a smaller effect (effect size −0.51; 95% CI: −0.84, −0.18). This discrepancy may result from differences in intervention protocols, study populations, and types of music used. In addition, the original studies on music therapy were limited in number, and future large-sample studies are needed to confirm its effects. Manual therapies, including acupressure, acupuncture, massage therapy, and aromatherapy, consistently showed positive effects in four meta-analyses, with an average effect size of 1.00. These results suggest that manual therapy is effective in improving sleep quality among older adults, but due to the lack of blinding in the original studies, the quality of evidence was rated as very low. Further refinement in study design is needed in the future. In addition, one meta-analysis on joint therapy reported a significant effect size of 1.18, indicating that the combination of multiple non-pharmacological interventions has synergistic effects and highlights the potential of integrated approaches in improving sleep.

Overall, the consistent positive results highlight the value of non-pharmacological interventions in sleep management for older adults. It is recommended that these therapies be considered in clinical practice. Future studies should aim to optimize these interventions and validate the findings through high-quality, large-scale trials. This umbrella review focused on RCTs to obtain the highest level of evidence. Although there are other types of non-pharmacological interventions, such as light therapy, they were not included in this analysis because the included studies were not RCTs, which helped ensure the quality of evidence. This review adopted a comprehensive search strategy, with study selection and data extraction conducted in duplicate. The degree of study overlap was only 3.68% (slight overlap). In addition, we could not find any previously published umbrella review on this topic for comparison.

Any umbrella review is subject to the limitations of the included systematic reviews and meta-analyses. Although the systematic reviews included here covered a wide range of clinical populations, these populations were not exhaustive. Using the AMSTAR 2 tool, only a few reviews were rated as high confidence, although this result is consistent with reports in other areas of medicine (42, 43). Very few meta-analyses analyzed the long-term effects of non-pharmacological interventions. More long-term follow-up studies are needed in the future to clarify the lasting effects of non-pharmacological interventions.

### Limitations

There are some limitations in the current body of evidence. First, the original studies included in this umbrella review showed significant differences in populations, which may reduce the stability and comparability of effect size estimates. Additionally, only English-language literature was included. Although most randomized controlled trials were conducted in China, the lack of regional diversity may affect the comprehensiveness of the findings. Future research should expand the language scope of the included literature to enhance the representativeness of the results. Second, this review did not include systematic reviews of sleep interventions targeting cognitively impaired populations. While non-pharmacological interventions have been tested to improve sleep in cognitively impaired patients, most lack robust evidence to guide clinical practice. The limited number of such interventions for older adults with cognitive impairment means that the quantity and quality of systematic reviews are insufficient. Furthermore, significant heterogeneity in inclusion criteria, outcome measures, and interventions complicates the integration of these studies with general population research. Further studies are needed to address these gaps. Regarding outcome assessment, most studies relied on subjective scales or sleep diaries, which may not align with objective measurements, such as polysomnography (PSG), potentially reducing accuracy. This review also did not specify the time points for sleep-related outcomes, which could affect the interpretation of the results. Moreover, there is a lack of comparative studies on the effects of different types of exercise therapy. Future research should address this issue. Finally, this study focused on behavioral/psychological and exercise-psychophysiological interventions (e.g., cognitive behavioral therapy, exercise, Tai Chi), excluding neuromodulation techniques that involve devices or procedures. This may have led to the omission of relevant evidence on such techniques. Future research should explore the combined effects of non-pharmacological interventions on sleep and identify the most effective components within multifaceted programs.

## Conclusion

Non-pharmacological interventions show positive effects in improving sleep-related indicators in older adults. This umbrella review suggests that CBT, Mindfulness Therapy, and Exercise Therapy are particularly recommended among various non-pharmacological interventions. In particular, Exercise Therapy stands out as especially practical due to its cost-effectiveness and convenience. However, since this study involved various subgroups of older adults, their baseline characteristics, health status and intervention effects may vary. Therefore, caution is needed regarding the generalizability of the study’s conclusions and their applicability in specific contexts. Although the effects of different non-pharmacological interventions on sleep are of clinical importance, it is worth noting that the benefits of these interventions are mostly concentrated in the short term, i.e., they are observed immediately after the intervention. Further research should focus on the long-term effects of these interventions and how to optimize intervention protocols to maintain and enhance their long-term efficacy. Moreover, exploring the combined application of different intervention modalities and developing personalized treatment programs are also important future directions. In summary, although the quality of evidence is relatively low, non-pharmacological interventions have significant clinical application potential in improving sleep among older adults and can provide strong support for related clinical practices and health policy formulation as complementary therapies.

## Data Availability

The original contributions presented in the study are included in the article/Supplementary material, further inquiries can be directed to the corresponding authors.
