# Supplementary material for: Non-pharmacological interventions for sleep in older adults: an umbrella review and evidence map of randomized controlled trials
Source: Front Neurol. 2025 Sep 2;16:1655192. doi: 10.3389/fneur.2025.1655192 (PMC12439529; doi:10.3389/fneur.2025.1655192)
Supplement: Supplementary file 1 [file Table_1.docx]

**Supplementary Contents**

Table S1. Search strategy

Table S2. Excluded studies after full-text assessment

Table S3. The details of each type of non-pharmacological interventions

Table S4. AMSTAR2 scores of included systematic reviews

Table S5. Criteria evidence of GRADE(Demurtas et al., 2020)

Table S1. Search strategy

| Total (Searched by 2024.07.06, Items found: 1700) | |
| --- | --- |
| 1. Pubmed (Searched by 2024.07.06, Items found: 354) | |
| #1 | "Aged"[Mesh] OR "Geriatrics"[Mesh] OR "aged"[Title/Abstract] OR "elderly"[Title/Abstract] OR "older"[Title/Abstract] OR "older people"[Title/Abstract] OR "older adults"[Title/Abstract] OR "senior*"[Title/Abstract] OR "geriatric*"[Title/Abstract] |
| #2 | "Sleep"[Mesh] OR "Sleep Wake Disorders"[Mesh] OR "Sleep Initiation and Maintenance Disorders"[Mesh] OR "Sleep Deprivation"[Mesh] OR "sleep"[Title/Abstract] OR "Sleep Wake Disorders"[Title/Abstract] OR "Sleep Initiation and Maintenance Disorders"[Title/Abstract] OR "Sleep Deprivation"[Title/Abstract] OR "sleep disorder"[Title/Abstract] OR "sleep disorders"[Title/Abstract] OR "sleep disturbance"[Title/Abstract] OR "sleep disturbances"[Title/Abstract] OR "sleep fragmentation"[Title/Abstract] OR "insomnia*"[Title/Abstract] OR "hypersomnia"[Title/Abstract] OR "sleep healthy"[Title/Abstract] OR "sleep hygiene"[Title/Abstract] OR "sleep quality"[Title/Abstract] |
| #3 | "Exercise"[Mesh] OR "exercise"[Title/Abstract] OR "training"[Title/Abstract] OR "physical*"[Title/Abstract] OR "yoga"[Title/Abstract] OR "tai chi"[Title/Abstract] OR "Baduanjin"[Title/Abstract] OR "Strength Training"[Title/Abstract] OR "Endurance Training"[Title/Abstract] OR "Resistance Training"[Title/Abstract] |
| #4 | "Cognitive Behavioral Therapy"[Mesh] OR "Mindfulness" [Mesh] OR "cognitive behavioral therapy"[Title/Abstract] OR "cognitive therapy"[Title/Abstract] OR "behavior therapy"[Title/Abstract] OR "CBT"[Title/Abstract] OR "cognitive behavi*"[Title/Abstract] OR "Mindfulness"[Title/Abstract] |
| #5 | "Music Therapy"[Mesh] OR "music*"[Title/Abstract] |
| #6 | "Massage"[Mesh] OR "massag*"[Title/Abstract] OR "acupressure"[Title/Abstract] |
| #7 | "Light"[MeSH] OR "light*"[Title/Abstract] |
| #8 | "Aromatherapy"[MeSH] OR “Aromatherapies”[Title/Abstract] OR “Aroma*”[Title/Abstract] |
| #9 | “Meditation”[MeSH] OR “Meditati*”[Title/Abstract] |
| #10 | “Mind-body therapies”[MeSH] OR “Mind Body”[Title/Abstract] OR “Mind-Body”[Title/Abstract] |
| #11 | #3 OR #4 OR #5 OR #6 OR #7 OR #8 OR #9 OR #10 |
| #12 | “Systematic Review”[Title/Abstract] OR “Meta-Analysis”[Title/Abstract] |
| #13 | #1 AND #2 AND #11 AND #12 |
| 1. Cochrane Library (Searched by 2024.07.06, Items found: 84) | |
| #1 | MeSH descriptor: [Aged] explode all trees |
| #2 | (aged):ti,ab,kw OR (older*):ti,ab,kw OR (senior*):ti,ab,kw OR (elderly):ti,ab,kw |
| #3 | #1 OR #2 |
| #4 | MeSH descriptor: [Sleep Wake Disorders] explode all trees |
| #5 | ("sleep disorder"):ti,ab,kw OR ("sleep disturbance"):ti,ab,kw OR (insomnia*):ti,ab,kw OR ("sleep quality"):ti,ab,kw |
| #6 | #4 OR #5 |
| #7 | MeSH descriptor: [Exercise] explode all trees OR MeSH descriptor: [Mindfulness] explode all trees OR MeSH descriptor: [Cognitive Behavioral Therapy] explode all trees OR MeSH descriptor: [Music Therapy] explode all trees OR MeSH descriptor: [Massage] explode all trees OR MeSH descriptor: [Aromatherapy] explode all trees OR MeSH descriptor: [Meditation] explode all trees OR MeSH descriptor: [Mind-body therapies] explode all trees |
| #8 | (exercise):ti,ab,kw OR ("cognitive behavioral therapy"):ti,ab,kw OR (mindfulness):ti,ab,kw OR (music):ti,ab,kw OR (massag*):ti,ab,kw OR (light):ti,ab,kw OR (acuputure):ti,ab,kw OR (aromatherapy):ti,ab,kw OR (meditati*):ti,ab,kw OR (mind-body):ti,ab,kw |
| #9 | #7 OR #8 |
| #10 | MeSH descriptor: [Systematic Reviews as Topic] explode all trees OR MeSH descriptor: [Meta Analysis as Topic] explode all trees |
| #11 | (“systematic review”):ti,ab,kw OR (meta-analysis):ti,ab,kw |
| #12 | #10 OR #11 |
| #13 | #3 AND #6 AND #9 AND #12 |
| 1. Embase (Searched by 2024.07.06, Items found: 693) | |
| #1 | 'aged'/exp |
| #2 | aged:ab,ti OR elderly:ab,ti OR older*:ab,ti OR senior*:ab,ti |
| #3 | #1 OR #2 |
| #4 | 'sleep disorder'/exp |
| #5 | 'sleep disorder*':ab,ti OR 'sleep disturbance*':ab,ti OR insomnia*:ab,ti OR 'sleep quality':ab,ti OR 'sleep healthy':ab,ti |
| #6 | #4 OR #5 |
| #7 | 'exercise'/exp OR 'cognitive behavioral therapy'/exp OR 'music therapy'/exp OR 'massage'/exp OR 'light'/exp OR 'Aromatherapy'/exp OR 'Meditation'/exp OR 'Mind-body therpies'/exp |
| #8 | exercise:ab,ti OR physical*:ab,ti OR yoga:ab,ti OR 'tai chi':ab,ti OR baduanjin:ab,ti OR 'endurance training':ab,ti OR 'resistance training':ab,ti OR 'cognitive behavioral therapy':ab,ti OR 'cognitive therapy':ab,ti OR 'behavior therapy':ab,ti OR cbt:ab,ti OR 'cognitive behavi*':ab,ti OR mindfulness:ab,ti OR music:ab,ti OR massag*:ab,ti OR light:ab,ti OR acuputure:ab,ti OR Aromatherapy:ab,ti OR Meditati*:ab,ti OR Mind-body:ab,ti |
| #9 | #7 OR #8 |
| #10 | 'systematic review'/exp OR 'meta analysis'/exp OR 'systematic review':ab,ti OR 'meta analysis':ab,ti |
| #11 | #3 AND #6 AND #9 AND #10 |
| 1. CINAHL (Searched by 2024.07.06, Items found: 77) | |
| #1 | TI aged OR TI elderly OR TI older* OR TI senior* |
| #2 | AB aged OR AB elderly OR AB older* OR AB senior* |
| #3 | #1 OR #2 |
| #4 | TI sleep disorder* OR TI sleep disturbance* OR TI insomnia* OR TI sleep quality |
| #5 | AB sleep disorder* OR AB sleep disturbance* OR AB insomnia* OR AB sleep quality |
| #6 | #4 OR #5 |
| #7 | TI exercise OR TI physical* OR TI yoga OR TI tai chi OR TI resistance training OR TI cognitive behavioral therapy OR TI massag* OR TI music therapy OR TI mindfulness OR TI light OR TI acuputure OR TI Aromatherapy OR TI Meditati* OR TI Mind-body |
| #8 | AB exercise OR AB physical* OR AB yoga OR AB tai chi OR AB resistance training OR AB cognitive behavioral therapy OR AB massag* OR AB music therapy OR AB mindfulness OR AB light OR AB acuputure OR AB Aromatherapy OR AB Meditati* OR AB Mind-body |
| #9 | #7 OR #8 |
| #10 | TI systematic review OR TI meta analysis |
| #11 | AB systematic review OR AB meta analysis |
| #12 | #10 OR #11 |
| #13 | #3 AND #6 AND #9 AND #12 |
| 1. Scopus (Searched by 2024.07.06, Items found: 430) | |
| #1 | TITLE-ABS-KEY(old OR older* OR aged OR senior* OR elder*) |
| #2 | TITLE-ABS-KEY("sleep disorder*" OR "sleep disturbance*" OR "insomnia*" OR "sleep quality") |
| #3 | TITLE-ABS-KEY("exercise" OR "physical*" OR "yoga" OR "tai chi" OR "resistance training" OR "cognitive behavioral therapy" OR "massag*" OR "music therapy" OR "mindfulness" OR "light" OR "acuputure" OR "aromatherapy" OR "Meditati*" OR "Mind-body") |
| #4 | TITLE ("Meta-Analysis" OR "Systematic review") |
| #5 | #1 AND #2 AND #3 AND #4 |
| 1. PsycINFO (Searched by 2024.07.06, Items found: 62) | |
| #1 | mainsubject.Exact(Aged OR Aging) OR tiab(old OR older* OR aged OR senior* OR elder OR elderly) |
| #2 | mainsubject.Exact("sleep disorder" OR "sleep disturbance" OR "insomnia" OR "sleep quality") OR tiab("sleep disorder*" OR "sleep disturbance*" OR "insomnia*" OR "sleep quality") |
| #3 | mainsubject.Exact("cognitive behavioral therapy" OR "aromatherapy" OR "light" OR "meditation" OR "mind-body therapies" OR "exercise" OR "music therapy" OR "massage") OR tiab("exercise" OR "physical*" OR "yoga" OR "tai chi" OR "resistance training" OR "cognitive behavioral therapy" OR "massag*" OR "music therapy" OR "mindfulness" OR "light" OR "acuputure" OR "aromatherapy" OR "Meditati*" OR "Mind-body") |
| #4 | mainsubject.Exact("Meta-Analysis" OR "Systematic review") OR tiab("Meta-Analysis" OR "Systematic review") |
| #5 | #1 AND #2 AND #3 AND #4 |

Table S2. Excluded studies after full-text assessment

| **No.** | **Reference** | **Reason** |
| --- | --- | --- |
| 2 | Montgomery, Paul, and Jane Dennis. "A systematic review of non-pharmacological therapies for sleep problems in later life." Sleep medicine reviews 8.1 (2004): 47-62. | No meta-analysis conducted |
| 3 | De Niet, Gerrit, et al. "Music‐assisted relaxation to improve sleep quality: meta‐analysis." Journal of advanced nursing 65.7 (2009): 1356-1364. | Mean age of participants less than 60 years |
| 4 | Horton, M. S., M. Pugh, and V. A. Lawrence. "Nonpharmacological Therapy for Insomnia in Elders: A Systematic." JOURNAL OF THE AMERICAN GERIATRICS SOCIETY. Vol. 58. COMMERCE PLACE, 350 MAIN ST, MALDEN 02148, MA USA: WILEY-BLACKWELL PUBLISHING, INC, 2010. | Full text unavailable |
| 5 | Chambe, J., et al. "Efficacy of light therapy in insomnia: a systematic review." Journal of Sleep Research. Vol. 21. 111 RIVER ST, HOBOKEN 07030-5774, NJ USA: WILEY-BLACKWELL, 2012. | Full text unavailable |
| 7 | Alessi, Cathy, and Michael V. Vitiello. "Insomnia (primary) in older people: non-drug treatments." BMJ clinical evidence 2015 (2015). | Not all included studies are RCTs |
| 9 | Geiger-Brown, Jeanne M., et al. "Cognitive behavioral therapy in persons with comorbid insomnia: a meta-analysis." Sleep medicine reviews 23 (2015): 54-67. | Mean age of participants less than 60 years |
| 10 | Ho, Fiona Yan-Yee, et al. "Self-help cognitive-behavioral therapy for insomnia: a meta-analysis of randomized controlled trials." Sleep medicine reviews 19 (2015): 17-28. | Mean age of participants less than 60 years |
| 11 | Koffel, Erin A., Jonathan B. Koffel, and Philip R. Gehrman. "A meta-analysis of group cognitive behavioral therapy for insomnia." Sleep medicine reviews 19 (2015): 6-16. | Mean age of participants less than 60 years |
| 12 | Wang, Claudia, Dong-Chul Seo, and Hongtao Li. "Tai Chi as an Intervention on Health Promotion for Older Adults: A Systematic Review." Integrative Medicine Research 4.1 (2015): 117. | Not written in English |
| 14 | Spiewak, Christina, et al. "A Systematic Review of the Outcomes of Therapeutic Yoga With Older Adults." The American Journal of Occupational Therapy 71.4_Supplement_1 (2017): 7111505134p1-7111505134p1. | Full text unavailable |
| 15 | Brasure, Michelle, et al. "Psychological and behavioral interventions for managing insomnia disorder: an evidence report for a clinical practice guideline by the American College of Physicians." Annals of internal medicine 165.2 (2016): 113-124. | Mean age of participants less than 60 years |
| 16 | Guo, Yucheng, et al. "Health benefits of traditional Chinese sports and physical activity for older adults: a systematic review of evidence." Journal of Sport and Health Science 5.3 (2016): 270-280. | No meta-analysis conducted |
| 17 | Hwang, Eunhee, and Sujin Shin. "Effectiveness of non-pharmacological intervention for insomnia: a systematic review and meta-analysis." Indian Journal of Science and Technology 9.40 (2016): 1-9. | Not all included studies are RCTs |
| 18 | Shergis, Johannah Linda, et al. "A systematic review of acupuncture for sleep quality in people with insomnia." Complementary therapies in medicine 26 (2016): 11-20. | Mean age of participants less than 60 years |
| 19 | Kim, Ji Hyun, and Pok Ja Oh. "Effects of non-pharmacological interventions on primary insomnia in adults aged 55 and above: a meta-analysis." Korean Journal of Adult Nursing 28.1 (2016): 13-29. | Full text unavailable |
| 20 | Smallfield, Stacy, and Whitney Lucas Molitor. "Occupational therapy interventions addressing sleep for community-dwelling older adults: A systematic review." The American Journal of Occupational Therapy 72.4 (2018): 7204190030p1-7204190030p9. | No meta-analysis conducted |
| 21 | Van Straten, Annemieke, et al. "Cognitive and behavioral therapies in the treatment of insomnia: a meta-analysis." Sleep medicine reviews 38 (2018): 3-16. | Mean age of participants less than 60 years |
| 22 | Waits, Alexander, et al. "Acupressure effect on sleep quality: a systematic review and meta-analysis." Sleep medicine reviews 37 (2018): 24-34. | Mean age of participants less than 60 years |
| 24 | Lin, Pi-Chu, et al. "Effects of aromatherapy on sleep quality: A systematic review and meta-analysis." Complementary therapies in medicine 45 (2019): 156-166. | Mean age of participants less than 60 years |
| 25 | Roozbeh, Nasibeh, et al. "Effect of lavender on sleep, sexual desire, vasomotor, psychological and physical symptom among menopausal and elderly women: A systematic review." Journal of menopausal medicine 25.2 (2019): 88-93. | No meta-analysis conducted |
| 26 | Shang, Binghan, et al. "Nonpharmacological interventions to improve sleep in nursing home residents: A systematic review." Geriatric Nursing 40.4 (2019): 405-416. | No meta-analysis conducted |
| 27 | Shohani, Masoumeh, et al. "Assessing The Quality of Sleep in Iranian Elderly People by the Standard Pittsburgh Sleep Quality Index: Systematic Review and Meta-Analysis Method." Indian Journal of Forensic Medicine & Toxicology 13.4 (2019). | Not all included studies are RCTs |
| 28 | Benz, Fee, et al. "The efficacy of cognitive and behavior therapies for insomnia on daytime symptoms: A systematic review and network meta-analysis." Clinical Psychology Review 80 (2020): 101873. | Mean age of participants less than 60 years |
| 30 | Vanderlinden, J., F. Boen, and J. G. Z. Van Uffelen. "Effects of physical activity programs on sleep outcomes in older adults: a systematic review." International Journal of Behavioral Nutrition and Physical Activity 17 (2020): 1-15. | Not all included studies are RCTs |
| 32 | Her, Jihoo, and Mi-Kyoung Cho. "Effect of aromatherapy on sleep quality of adults and elderly people: A systematic literature review and meta-analysis." Complementary therapies in medicine 60 (2021): 102739. | Not all included studies are RCTs |
| 33 | Richter, Kneginja, et al. "Cognitive behavioral treatment of insomnia in the elderly—when and how to treat?." Somnologie 22 (2018): 245-250. | Not written in English |
| 34 | Lee, Seonheui, and Soyoung Yu. "Effectiveness of information and communication technology (ICT) interventions in elderly’s sleep disturbances: A systematic review and meta-analysis." Sensors 21.18 (2021): 6003. | Not all included studies are RCTs |
| 35 | Li, Shanshan, et al. "Effect of exercise intervention on primary insomnia: a meta-analysis." The Journal of Sports Medicine and Physical Fitness 61.6 (2021): 857-866. | Full text unavailable |
| 36 | Petrovsky, Darina V., et al. "Effects of music interventions on sleep in older adults: A systematic review." Geriatric Nursing 42.4 (2021): 869-879. | Not all included studies are RCTs |
| 37 | Tang, Yueheng, et al. "The therapeutic effect of aromatherapy on insomnia: a meta-analysis." Journal of affective disorders 288 (2021): 1-9. | Mean age of participants less than 60 years |
| 38 | Wang, Cong, et al. "Effects of music intervention on sleep quality of older adults: a systematic review and meta-analysis." Complementary therapies in medicine 59 (2021): 102719. | Not all included studies are RCTs |
| 39 | Wang, Feifei, and Szilvia Boros. "The effect of physical activity on sleep quality: a systematic review." European journal of physiotherapy 23.1 (2021): 11-18. | Not all included studies are RCTs |
| 44 | Ko, Ling-Hsin, et al. "Effects of health qigong on sleep quality: A systematic review and meta-analysis of randomized controlled trials." Complementary Therapies in Medicine 71 (2022): 102876. | Mean age of participants less than 60 years |
| 45 | Luan, Xin, Xiaodan Zhang, and Yixin Zhou. "[Retracted] The Role and Clinical Observation of Traditional Chinese Medicine in Relieving Senile Insomnia: A Systematic Review and Meta‐Analysis." BioMed Research International 2022.1 (2022): 9484095. | Full text unavailable |
| 46 | Luo, Shu-Wen, et al. "A systematic review and meta-analysis of acupuncture combined with Tuina in the treatment of insomnia." Medicine 101.51 (2022): e30703. | Mean age of participants less than 60 years |
| 47 | Jung, Sun Ok, Hyeyoung Kim, and Eunju Choi. "The Effects of Non-pharmacological Interventions on Sleep among Older Adults in Korean Long-term Care Facilities: A Systematic Review and Meta-analysis." Journal of Korean Academy of Community Health Nursing 33.3 (2022): 340-355. | Not all included studies are RCTs |
| 48 | Paez, A., et al. "The effectiveness of exercise interventions targeting sleep in older adults: a systematic review and meta-analysis." JOURNAL OF SLEEP RESEARCH. Vol. 31. 111 RIVER ST, HOBOKEN 07030-5774, NJ USA: WILEY, 2022. | Full text unavailable |
| 49 | Yang, Jiayi, et al. "Mindfulness-Based Movement Intervention to improve sleep quality: A meta-analysis and moderator analysis of randomized clinical trials." International Journal of Environmental Research and Public Health 19.16 (2022): 10284. | Mean age of participants less than 60 years |
| 50 | Amani, Omid, et al. "Effectiveness of internet-delivered cognitive behavioral therapy for insomnia: a systematic review and meta-analysis." Biological Rhythm Research 54.10 (2023): 647-663. | Age not reported |
| 51 | Bandyopadhyay, Nita, et al. "Effects of Yogic Intervention on sleep quality of healthy elderly: A systematic review." Universal Journal of Public Health 11.1 (2023): 78-88. | Not all included studies are RCTs |
| 53 | Deng, Wenrui, et al. "eHealth-based psychosocial interventions for adults with insomnia: systematic review and meta-analysis of randomized controlled trials." Journal of Medical Internet Research 25 (2023): e39250. | Mean age of participants less than 60 years |
| 54 | Eun, Hong-Bum, et al. "Effect of Exercise on Sleep in the Middle-aged and Older adult: A Systematic Review and Meta-Analysis of Randomized Controlled Trials." Exercise Science 32.1 (2023): 21-32. | Not written in English |
| 57 | Sella, Enrico, et al. "Non-pharmacological interventions targeting sleep quality in older adults: a systematic review and meta-analysis." Aging & Mental Health 27.5 (2023): 847-861. | Not all included studies are RCTs |
| 60 | Gao, Xin, et al. "Effects of different types of exercise on sleep quality based on PSQI in middle-aged and older adults: a network meta-analysis." Journal of Clinical Sleep Medicine (2024): jcsm-11106. | Full text unavailable |
| 61 | Khaleghi, M. M., and F. Ahmadi. "Effect of different exercises on sleep quality in elderly women: a systematic review." Comparative Exercise Physiology 1.aop (2024): 1-12. | No meta-analysis conducted |
| 65 | Wang, Zhen, et al. "Effect of tuina on sleep quality, psychological state and neurotransmitter level in patients with insomnia: a systematic review and meta-analysis." Frontiers in Neurology 15 (2024): 1273194. | Mean age of participants less than 60 years |

Table S3. The details of each type of non-pharmacological interventions

| **Types of non-pharmacological interventions** | **Details of interventions** |
| --- | --- |
| 1. Cognitive behavior therapy | Cognitive Behavioral Therapy (CBT) primarily addresses the long-term maintenance factors of insomnia. By correcting patients' misconceptions about sleep and establishing structured sleep behaviors, CBT fundamentally resolves the issues causing insomnia. Therefore, CBT can be used for insomnia regardless of its triggering factors. The forms of CBT are diverse and mainly include five components: sleep hygiene education, stimulus control, sleep restriction, relaxation therapy, and cognitive therapy. Consequently, in this review, relaxation therapy, nurse intervention, and sleep hygiene therapy are also categorized under cognitive behavioral therapy. |
| 1. Mindfulness therapy | Mindfulness therapy, also known as mindfulness-based therapy, is a therapeutic approach that incorporates mindfulness practices to help individuals manage various mental health conditions and improve overall well-being. |
| 1. Exercise therapy | Exercise therapy is a treatment method that improves health and function through exercise and physical activity. It is used to prevent and treat various physical and psychological issues, promoting overall well-being. Exercise therapy typically includes the following types: aerobic exercises; strength training; flexibility exercises: such as yoga and stretching exercises; and balance and coordination exercises: such as Tai Chi and specific balance training. |
| 1. Music therapy | Music therapy is a therapeutic approach that uses music to improve physical, emotional, cognitive, and social functioning. It includes active music therapy, passive music therapy, and music improvisation. |
| 1. Manual therapy | Manual therapy commonly includes acupressure, acupuncture, massage therapy, and aromatherapy. Acupressure is a method that involves applying pressure to specific points on the body for therapeutic purposes. Typically, fingers, palms, elbows, or specialized tools are used to apply the pressure, aiming to relieve pain, reduce stress, and improve overall health. Acupuncture is a traditional Chinese medicine technique that involves inserting fine needles into specific points on the body. The purpose is to balance the body's energy flow (Qi) to promote healing, alleviate pain, and improve various health conditions. Massage therapy manipulates the muscles and soft tissues of the body through techniques such as kneading, stroking, and applying pressure. It aims to reduce muscle tension, improve blood circulation, promote relaxation, and alleviate various physical discomforts. Aromatherapy uses essential oils extracted from plants for therapeutic purposes. These oils can be inhaled, applied to the skin during massage, or added to baths. The goal of aromatherapy is to enhance psychological and physical well-being by reducing stress, improving mood, and promoting relaxation. |
| 1. Joint interventions | Refers to greater than or equal to two non-pharmacological interventions, such as cognitive behavior therapy, mindfulness therapy, exercise therapy, music therapy and manual therapy. |

Table S4. AMSTAR2 scores of included systematic reviews

| Item | Q1 | Q2 | Q3 | Q4 | Q5 | Q6 | Q7 | Q8 | Q9 | Q10 | Q11 | Q12 | Q13 | Q14 | Q15 | Q16 | Overall |
| --- | --- | --- | --- | --- | --- | --- | --- | --- | --- | --- | --- | --- | --- | --- | --- | --- | --- |
| Montgomery P, et al. 1996 | YES | YES | NO | YES | YES | YES | YES | YES | YES | NO | YES | YES | YES | YES | YES | YES | Moderate |
| Yang P Y, et al. 2012 | YES | NO | NO | YES | YES | YES | YES | YES | NO | NO | YES | YES | YES | NO | NO | NO | Critically Low |
| Du S, et al. 2015 | YES | NO | NO | YES | YES | YES | YES | YES | YES | NO | YES | YES | YES | YES | YES | YES | Low |
| Wu W, et al. 2015 | YES | NO | NO | YES | YES | YES | YES | YES | YES | NO | YES | YES | YES | YES | YES | YES | Low |
| He B, et al. 2019 | YES | NO | NO | Partially YES | YES | YES | YES | YES | YES | NO | YES | YES | YES | NO | YES | YES | Low |
| Chen M C, et al. 2020 | NO | NO | NO | YES | YES | YES | Partially YES | YES | YES | NO | YES | YES | YES | YES | YES | YES | Low |
| Chen C T, et al. 2021 | YES | NO | NO | YES | YES | YES | YES | YES | YES | NO | YES | YES | YES | YES | YES | YES | Low |
| He W, et al. 2021 | YES | NO | NO | YES | YES | YES | YES | YES | YES | NO | YES | YES | YES | YES | YES | YES | Low |
| Dincer B, et al. 2022 | YES | YES | YES | YES | YES | YES | YES | YES | YES | NO | YES | YES | YES | YES | YES | YES | High |
| Hasan F, et al. 2022 | YES | YES | NO | YES | YES | YES | YES | YES | YES | NO | YES | YES | YES | YES | NO | YES | Low |
| Huang K, et al. 2022 | YES | YES | NO | YES | YES | YES | YES | YES | YES | NO | YES | YES | YES | YES | YES | YES | Moderate |
| Chen Y C, et al. 2023 | YES | YES | YES | YES | YES | YES | NO | YES | YES | NO | YES | YES | YES | YES | YES | YES | Low |
| González-Martín A M, et al. 2023 | YES | NO | NO | YES | YES | YES | YES | YES | YES | NO | YES | YES | YES | NO | YES | YES | Low |
| Gu H J, et al. 2023 | YES | NO | NO | YES | YES | YES | YES | YES | YES | NO | YES | YES | YES | YES | YES | YES | low |
| Solis-Navarro L, et al. 2023 | YES | YES | NO | YES | YES | YES | YES | YES | YES | NO | YES | YES | YES | YES | YES | YES | Moderate |
| Chang H, et al. 2024 | YES | YES | NO | YES | YES | YES | YES | YES | YES | NO | YES | YES | YES | NO | NO | YES | Low |
| Lannon-Boran C, et al. 2024 | YES | YES | NO | YES | YES | YES | YES | YES | YES | NO | YES | YES | YES | YES | YES | YES | Moderate |
| Li L, et al. 2024 | YES | YES | NO | YES | YES | YES | Partially YES | YES | YES | NO | YES | YES | YES | YES | YES | YES | Moderate |
| Lyu L, et al. 2024 | YES | YES | NO | YES | YES | YES | YES | YES | YES | NO | YES | YES | YES | YES | YES | YES | Moderate |

Q1: Did the research questions and inclusion criteria for the review include the components of PICO?

Q2: Did the report of the review contain an explicit statement that the review methods were established prior to the conduct of the review and did the report justify any signifcant deviations from the protocol?

Q3: Did the review authors explain their selection of the study designs for inclusion in the review?

Q4: Did the review authors use a comprehensive literature search strategy?

Q5: Did the review authors perform study selection in duplicate?

Q6: Did the review authors perform data extraction in duplicate?

Q7: Did the review authors provide a list of excluded studies and justify the exclusions?

Q8: Did the review authors describe the included studies in adequate detail?

Q9: Did the review authors use a satisfactory technique for assessing the risk of bias (RoB) in individual studies that were included in the review?

Q10: Did the review authors report on the sources of funding for the studies included in the review?

Q11: If meta-analysis was performed, did the review authors use appropriate methods for statistical combination of results?

Q12: If meta-analysis was performed, did the review authors assess the potential impact of RoB in individual studies on the results of the meta-analysis or other evidence synthesis?

Q13: Did the review authors account for RoB in individual studies when interpreting/discussing the results of the review?

Q14: Did the review authors provide a satisfactory explanation for, and discussion of, any heterogeneity observed in the results of the review?

Q15: If they performed quantitative synthesis, did the review authors carry out an adequate investigation of publication bias (small study bias) and discuss its likely impact on the results of the review?

Q16: Did the review authors report any potential sources of confict of interest, including any funding they received for conducting the review?

**Critical domains**: Q2, Q4, Q7, Q9, Q11, Q13, and Q15.

High: No or one non-critical weakness.

Moderate: More than one non-critical weakness.

Low: One critical few with or without non-critical weaknesses.

Critically low: More than one critical few with or without non-critical weakness

Table S5. Criteria evidence of GRADE(Demurtas et al., 2020)

| Downgrade | Risk of bias | Inconsistency | Indirectness | Imprecision | Publication Bias |
| --- | --- | --- | --- | --- | --- |
| -1 | If 1 or more of the 3 criteria  (randomization, masking,  dropout rate ≤30%) is not met in  10-30% of trials included in the  systematic review | I^2^ 50-74% | The question being addressed by  the guideline panel is different  from the available evidence  regarding the PICO or regarding  the characteristics of those who  will deliver the intervention | (a) The overall number of  individuals included in trials is  low (less than 400 individuals,  both treatment arms) OR (b) the  95% confidence interval includes  both 1) no effect and 2)  appreciable benefit (RR: ≤0.75) or  appreciable harm (RR: ≥1.25)* | - |
| -2 | If 1 or more of the 3 criteria  (randomization, masking,  dropout rate ≥30%) is not met in  >30% of trials included in the  systematic review | I^2^≥75% | The question being addressed by  the guideline panel is markedly  different from the available  evidence regarding the PICO or  regarding the characteristics of  those who will deliver the  intervention | (a) the overall number of  individuals included in trials is  very low (fewer than 400  individuals, both treatment arms)  AND (b) the 95% confidence  interval includes both 1) no effect  and 2) appreciable benefit (RR:  ≤0.75) or appreciable harm  (RR: ≥1.25)* | Egger’s test  (P value)＜.05 |

PICO, Population, Intervention, Comparison, and Outcomes.

For dichotomous outcomes, “no effect” means an estimate with a confidence interval that crosses 1; appreciable benefit or appreciable harm means that the upper or lower confidence limit crosses a risk of 1.25 or 0.75.

*For continuous outcomes, “no effect” means an SMD with a confidence interval that crosses zero; appreciable benefit or appreciable harm means that the upper or lower confidence limit crosses an effect size of 0.5 in either direction.
